# Phytochemical Analysis and Antimicrobial, Antinociceptive, and Anti-Inflammatory Activities of Two Chemotypes of *Pimenta pseudocaryophyllus* (Myrtaceae)

**DOI:** 10.1155/2012/420715

**Published:** 2012-10-02

**Authors:** Joelma Abadia Marciano de Paula, Maria do Rosário Rodrigues Silva, Maysa P. Costa, Danielle Guimarães Almeida Diniz, Fabyola A. S. Sá, Suzana Ferreira Alves, Élson Alves Costa, Roberta Campos Lino, José Realino de Paula

**Affiliations:** ^1^Unit of Exact and Technologic Sciences, Goiás State University, Anápolis, Brazil; ^2^Institute of Tropical Pathology and Public Health, Federal University of Goiás, Goiânia, Brazil; ^3^Faculty of Pharmacy, Federal University of Goiás, Goiânia, Brazil; ^4^Department of Physiological Sciences, Institute of Biological Sciences, Federal University of Goiás, Goiânia, Brazil

## Abstract

Preparations from *Pimenta pseudocaryophyllus* (Gomes) L.R. Landrum (Myrtaceae) have been widely used in Brazilian folk medicine. This study aims to evaluate the antimicrobial activity of the crude ethanol extracts, fractions, semipurified substances, and essential oils obtained from leaves of two chemotypes of *P. pseudocaryophyllus* and to perform the antinociceptive and anti-inflammatory screening. The ethanol extracts were purified by column chromatography and main compounds were spectrally characterised (1D and 2D ^1^H and ^13^C NMR). The essential oils constituents were identified by GC/MS. The broth microdilution method was used for testing the antimicrobial activity. The abdominal contortions induced by acetic acid and the ear oedema induced by croton oil were used for screening of antinociceptive and anti-inflammatory activities, respectively. The phytochemical analysis resulted in the isolation of pentacyclic triterpenes, flavonoids, and phenol acids. The oleanolic acid showed the best profile of antibacterial activity for Gram-positive bacteria (31.2–125 *μ*g mL^−1^), followed by the essential oil of the citral chemotype (62.5–250 *μ*g mL^−1^). Among the semipurified substances, **Ppm5**, which contained gallic acid, was the most active for *Candida* spp. (31.2 *μ*g mL^−1^) and *Cryptococcus* spp. (3.9–15.6 *μ*g mL^−1^). The crude ethanol extract and fractions from citral chemotype showed antinociceptive and anti-inflammatory effects.

## 1. Introduction

*Pimenta pseudocaryophyllus* (Gomes) L.R. Landrum (Myrtaceae) is a plant popularly known in Brazil as *pau-cravo, louro-cravo, louro, craveiro, craveiro-do-mato*, *chá-de-bugre,* and *cataia* [1–6]. In folk medicine, the leaves have been used to produce a refreshing drink with calming, diuretic, and aphrodisiac properties, as well as to treat colds and their complications and digestive and menstrual problems [2, 4–6]. It is the only *Pimenta* species native to Brazil [1, 3], and recent studies have shown the occurrence of different chemotypes for this species; these are characterised, for example, by the predominance of citral or (*E*)-methyl isoeugenol in the essential oils [7].

Invasive infections caused by *Candida* spp. and *Cryptococcus neoformans* have increased significantly in recent years [8–11]. The cause of this rise is often related to immunodeficiency associated with transplantation [11] and acquired immunodeficiency syndrome (AIDS) [9], as well as the use of intravascular catheters [10], dialysis, and abusive use of glucocorticoids and broad-spectrum antibiotics [8]. The drugs available to treat these infections are often not selective, are toxic, or have narrow action spectra [12]; moreover, some species are resistant to antifungal agents [13].

In the pharmacotherapy of bacterial diseases, the use of antibiotics in recent decades has significantly reduced the incidence of many infectious diseases. On the other hand, the severe side effects from many of these substances and the emergence of multiresistant microorganisms have stimulated research on the development of new antibacterial agents that are more specific, effective, and safe [24, 25].

There is thus a consensus on the need for further research on new alternative treatments for bacterial and fungal infections. Efforts have been focused on investigating the antimicrobial properties of products from plants [12, 25]. In addition to extensive use in folk medicine in diseases related to the common cold, which often involve microbial and/or inflammatory processes, experimental data show that plants of the genus *Pimenta* (Myrtaceae) have antimicrobial potential [26, 27]. The essential oil of *P. pseudocaryophyllus* leaves collected in two geographical areas in the state of São Paulo was active against strains of *C. albicans, Escherichia coli, Pseudomonas aeruginosa,* and *Staphylococcus aureus* [4]. In a previous study, we described the antimicrobial activity of the crude ethanol extract of leaves of this species collected in the Brazilian Cerrado against Gram-positive bacteria and *Candida albicans* [28], but we have not performed phytochemical studies for the isolation and identification of substances accountable for this activity.

Moreover, scientific studies have shown that *Pimenta* species, widely used in folk medicine, have analgesic and anti-inflammatory activities and are nontoxic in typical dosages [29–32]. Thus, the aims of this work were to carry out the phytochemical study, evaluate the antimicrobial activity of the crude ethanol extracts, fractions, semipurified substances, and essential oils obtained from leaves of two chemotypes of *P. pseudocaryophyllus*, and perform the screening of the antinociceptive and anti-inflammatory activities.

## 2. Material and Methods

### 2.1. General Experimental Procedures

The ^1^H and ^13^C one-dimensional and two-dimensional NMR spectra (Heteronuclear Single Quantum Coherence (HSQC) and Heteronuclear Multiple Bond Correlation (HMBC)) were obtained using a Bruker Avance III-500 spectrometer running at 500 MHz (^1^H) and 125 MHz (^13^C), using deuterated chloroform (CDCl_3_) and deuterated dimethyl sulfoxide (DMSO-d_6_) as solvents for nonpolar and polar samples, respectively. Tetramethylsilane (TMS) was used as an internal reference standard for chemical shifts (*δ*, ppm), and in some cases, the peak from the solvent was used for reference.

Essential oil samples were analysed by GC/MS using a QP5050A instrument (Shimadzu, Kyoto, Japan) with a capillary column of CBP-5 melted silica (30 m × 0.25 mm × 0.25 *μ*m; 5% phenyl-methylpolysiloxane film) (Shimadzu, Kyoto, Japan). Additionally, helium was used with a flow rate of 1 mL min^−1^ as a carrier gas, and a thermal profile of 60°C to 240°C with a gradient of 3°C min^−1^ followed by a gradient of 10°C min^−1^ up to 280°C, keeping a 5 min isotherm, was used. The ionisation energy of the detector was kept at 70 eV, and the sample injection volume following dilution in hexane (~10%) was 0.5 *μ*L. The analysis was carried out in the scanning mode, with a 40–400 *m*/*z* mass interval and 1 : 5 injection ratio. The quantitative analysis was obtained by integrating the total ion chromatogram (TIC). The identification of the components was performed by comparing the mass and retention indices (RI) calculated using values for the mass and retention indices available in the literature [33]. The retention indices were calculated by coinjection with a mixture of hydrocarbons, C_8_–C_32_ (Sigma, MO, USA), applying the Van Den Dool and Kratz equation [34].

The mass spectra of the flavonoids were collected using a coupled LC/EM/EM: Varian 1200L (Walnut Creek, CA, USA) system with a quadrupole ion analyser and ionisation through electron impact, 70 eV in positive mode *m*/*z* [M + H]^+^. The *m*/*z* scanning spectrum was 100–900, and the ionisation chamber was kept at room temperature.

The HPLC was held on Waters equipment (MA, USA) equipped with quaternary pump, e2695 separation module, 2998 diode array detector (PDA), and Empower 2.0 data processing system. The Varian C-18 (250 × 4.6 mm) column was used at room temperature. The detection system used was monitored at 360 nm for detection of flavonoids. The injection volume was of 30 *μ*L and the run in isocratic mode was used as mobile phase 75% of A [acetonitrile/water (0.1% acetic acid)—90 : 10] and 25% of B [MeOH (0.1% acetic acid)] at a constant flow of 1 mL min^−1^. The maximal running time was 20 min. Quercitrin (Sigma) was used as reference standard. The samples were previously filtered through Millex (Millipore, MA, USA) membrane and the mobile phase in 0.45 *μ*m PVDF membrane (Millipore).

For analytical and preparative thin-layer chromatography (ATLC, PTLC), chromatography plates prepared with G60 F_254_ Vetec silica gel, or F_254_ silica plates manufactured by Merck, RJ, Brazil, were used. Mixtures of organic solvents with the proper polarities for each analysed fraction were used as mobile phases. For detection of the components, the plates were observed under UV light at 254 nm and 365 nm and developed by the spraying of sulphuric vanillin followed by heating or exposure to a solution of 1% ethylamine diphenyl borate in methanol (NP) [35]. 

The column chromatography used silica gel G60 0.05–0.2 mm (Vetec, Brazil) and Sephadex LH-20 (GE Healthcare, Uppsala, Sweden) as stationary phases. As mobile phases, mixtures of organic solvents in increasing order of polarity were used, according to the polarity profiles of the fractions undergoing the process.

### 2.2. Plant Material

The leaves of *P. pseudocaryophyllus* of the (*E*)-methyl isoeugenol and citral chemotypes were collected in São Gonçalo do Abaeté, MG, Brazil, in February 2008, at 18°20′58.4′′S, 45°55′23.4′′W, and 864 m altitude. These two chemotypes are the most common found in the region of São Gonçalo do Abaeté and therefore were selected for this study.

The plant material was identified by Professor Carolyn Elinore Barnes Proença, Ph.D., of the Universidade de Brasília, and a voucher specimen (UFG-27159) was deposited at the herbarium of the Universidade Federal de Goiás.

### 2.3. Preparation of Extracts and Fractions

Air-dried leaves were ground in a knife mill. The crude ethanol extract was obtained by maceration of the powdered material (1 : 5 w/v) of each chemotype in 95% EtOH (v/v) at room temperature, followed by filtration, and concentration on a rotary evaporator at a temperature below 40°C. The extracts were concentrated to a constant weight and named crude ethanol extract of (*E*)-methyl isoeugenol chemotype (EE_M_) and crude ethanol extract of citral chemotype (EE_C_).

Fifty grams of each extract (EE_M_ and EE_C_) were dissolved in 250 mL MeOH/H_2_O (7 : 3) and subjected to liquid/liquid partitioning with solvents of increasing polarity (hexane, dichloromethane, and ethyl acetate). The final MeOH/H_2_O residual was concentrated in a rotary evaporator for elimination of the MeOH, and the resulting aqueous fraction was lyophilised. Therefore, four fractions were obtained from the crude extract of each chemotype: the hexane fraction (HF_M_ and HF_C_), the dichloromethane fraction (DF_M_ and DF_C_), the ethyl acetate fraction (EAF_M_ and EAF_C_), and the aqueous fraction (AF_M_ and AF_C_).

For obtaining of essential oils, leaves from each chemotype were subjected to hydrodistillation for 3 h in a Clevenger apparatus. The essential oils were dried with anhydrous Na_2_SO_4_, packaged in amber glass vials, and stored at −20°C until use. They were named essential oil of the citral chemotype (EO_C_) and essential oil of the (*E*)-methyl isoeugenol chemotype (EO_M_).

### 2.4. Isolation of the Chemical Constituents

**HF_C_**

The HF_C_ (2.5 g) was fractionated on a silica gel column (1 : 40) eluted with a gradient of hexane-EtOAc (5–100%), EtOAc-MeOH (1 : 1), and MeOH (100%). Seventy-five 20 mL fractions were collected and evaluated by TLC [hexane-EtOAc mixtures (10–30%)], observed under 254/365 nm UV light, and detected with sulphuric vanillin reagent. Similar fractions were pooled, resulting in 14 new fractions (CH-1 to CH-14). Fractions CH-8 through CH-11 were rechromatographed on a silica gel column eluted isocratically with CH_2_Cl_2_-petroleum ether (7 : 3), resulting in **Ppc-1** (133.5 mg).

**DF_C_**

The DF_C_ (1.0 g) was fractionated on a silica gel column (1 : 40) and eluted with a gradient of CH_2_Cl_2_-petroleum ether (7 : 3 and 9 : 1), CH_2_Cl_2_-EtOAc (5–100%), EtOAc-MeOH (1 : 1), and MeOH (100%). One hundred and one 5 mL fractions were collected. Using TLC [mobile phases composed of mixtures of CH_2_Cl_2_-EtOAc (75 : 25, 80 : 20, and 95 : 5)] with 254/365 nm UV light, and sulphuric vanillin reagent for detection, the fractions were combined into 20 new fractions (CD-1 to CD-20). Fractions CD-9, CD-10, and CD-11 were fractionated on a silica gel column eluted isocratically with petroleum ether-EtOAc (90 : 10). The semipurified fractions were rechromatographed on a silica gel column eluted isocratically with petroleum ether-acetone (40 : 10), resulting in **Ppc-2** (22.6 mg).

**EAF_C_**

In a column packed with Sephadex LH-20, 1.5 g of EAF_C_ was subjected to chromatography, using MeOH (100%) as the mobile phase. Two hundred and sixty-one 10–20 mL fractions were collected. The fractions were evaluated by TLC [EtOAc-formic acid-acetic acid-H_2_O (100 : 11 : 11 : 26)], observed under 254/365 nm UV light and developed with sulphuric vanillin and NP reagents. Similar fractions were pooled, resulting in 12 new fractions (CEA-1 to CEA-12).

CEA-12 showed a single spot at *R*_*f*_ close to 1, resulting in **Ppc-3** (59.7 mg). Fractions CEA-7, CEA-8, and CEA-9 showed typical spots of flavonoids with similar *R*_*f*_ values, so they were gathered and rechromatographed on a Sephadex LH-20 column eluted isocratically with MeOH-H_2_O (1 : 1). From this column, 175 fractions were collected and lyophilised after MeOH evaporation, evaluated by TLC, and grouped into 17 fractions (CEA-A to CEA-Q). The CEA-N fraction was subjected to preparative TLC [EtOAc-formic acid-acetic acid-H_2_O (100 : 11 : 11 : 26)], resulting in **Ppc-4** (18.7 mg). CEA-K, CEA-L, and CEA-M were gathered and subjected to preparative TLC, and the semipurified fraction was rechromatographed on a Sephadex LH-20 column eluted with a gradient of acetone-H_2_O (1 : 1, 6 : 4, and 7 : 3). From this column, 93 1 mL fractions were collected and subjected to solvent evaporation, lyophilisation, and monitoring by TLC, which led to **Ppc-5** (73.2 mg).

**AF_C_**

The AF_C_ (1.2 g) was chromatographed on packed column with Sephadex LH-20 eluted with MeOH (100%). Approximately 180 fractions of 1–10 mL were collected. The fractions were evaluated by TLC [isobutanol-acetic acid-water (14 : 1 : 5)] and exposed with UV light and 1% FeCl_3_ in 0.5 M hydrochloric acid. Fractions with the same chromatographic profiles were pooled, resulting in 11 new fractions (AC-1 to AC-11). AC-9 was reactive to FeCl_3_, resulting in **Ppc-6** (83.1 mg).

**HF_M_**

The HF_M_ (2.5 g) was fractionated on a silica gel column (1 : 40) and eluted with 100% hexane-EtOAc (5–100%), EtOAc-MeOH (1 : 1), and MeOH (100%). One hundred 10 mL fractions were collected and evaluated by TLC [hexane- EtOAc (10–30%)], observed under UV light, and detected with sulphuric vanillin reagent, resulting in 14 new fractions (MH-1 to MH-14). Fractions MH-4 and MH-5 were rechromatographed on a silica gel column eluted isocratically with CH_2_Cl_2_-petroleum ether (7 : 3), resulting in **Ppm-1** (106.2 mg) and **Ppm-2** (75.8 mg). Fractions MH-9 and MH-10 were gathered and subjected to isocratic silica gel column chromatography [hexane-CH_2_Cl_2_-MeOH (10 : 10 : 1)], which resulted in **Ppm-3** (36.9 mg).

**DF_M_**

The low output from DF_M_ hindered the use of chromatography columns in isolating possible compounds. Thus, TLC of the DF_M_ was carried out with some of the semipurified fractions for *R*_*f*_ comparison to verify whether some of the previously isolated compounds could also be found in this fraction. Runs were performed with DF_M_, **Ppc-1**, **Ppm-1**, and **Ppm-2** in CH_2_Cl_2_-petroleum ether (7 : 3), DF_M_ and **Ppc-2** in petroleum ether-acetone (40 : 10), and DF_M_ and **Ppm-3** in hexane-CH_2_Cl_2_-MeOH (10 : 10 : 1).

**EAF_M_**

The EAF_M_ (1.2 g) was subjected to chromatography on a packed column with Sephadex LH-20 [MeOH (100%)]. Approximately 159 1-mL fractions were collected and analysed by TLC [EtOAc-formic acid-acetic acid-H_2_O (100 : 11 : 11 : 26)]. Based on observation in UV light and detection with NP reagent, the fractions were pooled into four new fractions (MEA-1 through MEA-4). Fractions MEA-1 and MEA-2 showed typical spots of flavonoids with yellow-orange and greenish-yellow fluorescence after spraying with NP and observation under 365 nm UV light, with similar *R*_*f*_ values; thus, they were pooled and rechromatographed on a Sephadex LH-20 column eluted with a sequence of acetone-H_2_O mixtures (1 : 1, 6 : 4, and 7 : 3). From this column, 99 fractions were collected and, after evaluation by TLC, resulted in **Ppm-4** (273.4 mg).

**AF_M_**

The AF_M_ (1.2 g) was subjected to chromatography in a packed column with Sephadex LH-20 [MeOH (100%)]. Approximately 117 1-mL fractions were collected. The fractions were monitored by TLC [isobutanol-acid acetic-water (14 : 1 : 5)] with use of the UV light and 1% FeCl_3_ in 0.5 M hydrochloric acid. Fractions with the same chromatographic profiles were pooled, resulting in nine new fractions, and one of the most reactive to the 1% FeCl_3_ was named **Ppm-5** (203.6 mg).

### 2.5. Antimicrobial Activity

The extracts, fractions, essential oils, citral (Sigma), and semipurified substances were subjected to the microdilution test in broth for determining the minimum inhibitory concentration (MIC) in sterile 96-well microplates with “U-”shaped wells, as recommended by the Clinical and Laboratory Standards Institute (CLSI) [36, 37]. The experiments were performed in duplicate and repeated twice, independently, except for the semipurified substances.

American Type Culture Collection (ATCC) standard strains and clinical isolates were used (Table 1); these are kept in the Laboratories for Bacteriology and Mycology at the Institute of Tropical Pathology and Public Health, Federal University of Goiás, Goiânia, GO, Brazil.

Prior to testing, to reactivate the microbial cultures, the bacteria were transferred to Casoy broth and incubated at 37°C for 24 h, then transferred to inclined Casoy agar, and incubated at 37°C for an additional 24 h. The fungi were transferred to Sabouraud dextrose agar and incubated at room temperature for 24 to 48 h (*Candida *spp.) or 48 to 72 h (*Cryptococcus* spp.).

The culture medium used in the antibacterial activity test was 2x Müeller Hinton broth (MH) and that used in the antifungal activity test was RPMI 1640. The samples were solubilised in 10% dimethyl sulfoxide (DMSO) and diluted in MH broth (antibacterial activity) to obtain a concentration of 2000 *μ*g mL^−1^ or in RPMI (antifungal activity) to obtain a concentration of 1000 *μ*g mL^−1^. The semipurified substances were used at concentrations according to the available amount of the substance. For the preparation of samples with essential oils, 0.02% Tween 80 was added. Vancomycin (Sigma; 32 *μ*g mL^−1^) and gentamicin (Sigma; 128 *μ*g mL^−1^) were used as controls for Gram-positive and Gram-negative bacteria, respectively, and itraconazole (Sigma) at an initial concentration of 16 *μ*g mL^−1^ was used as a control for fungi.

The microplates inoculated with bacteria were incubated at 35°C ± 2°C for 16–20 h and for 24 hours at this temperature for *Staphylococcus*. One hour before the end of the incubation period, each well received 20 *μ*L of 0.5% triphenyl tetrazolium chloride (TTC), and the microplates were reincubated for approximately 30 min. The appearance of reddish colour was considered as proof of bacterial growth. The microplates inoculated with fungi were incubated at room temperature for 48 h (*Candida *spp.) and 72 h (*Cryptococcus* spp.). Fungal growth was checked visually, and the MIC was defined as the lowest concentration (*μ*g mL^−1^) of the sample fully capable of inhibiting bacterial and fungal growth.

### 2.6. Antinociceptive and Anti-Inflammatory Activity

These experiments were approved by the Animal Ethics Committee of the Federal University of Goiás, Protocol number 146/2008. Male, young adult mice, weighing between 25 and 30 g, were transferred to the experimental room two days before the tests, kept in a light/dark cycle of 12 h at 22 ± 2°C in a noise-free facility, with water and food *ad libitum*. The food was taken away 12 h before the test, keeping the water available *ad libitum* at all times.

#### 2.6.1. Evaluation of Antinociceptive Activity by Testing Abdominal Contortions Induced by Acetic Acid

The evaluation of abdominal contortions induced by acetic acid was carried out according to Koster et al. [38] and Lapa et al. [39]. Test groups consisting of 10 mice per dose of extract, fraction, standard drug or vehicle were used. The mice from the different experimental groups received intraperitoneal injections of 1.2% acetic acid solution (v/v, 10 mL kg^−1^) 1 h after oral intake (gavage) of the extracts or fractions (in different doses) or indomethacin (standard drug) or vehicle (control). They were then placed under glass funnels and the contortions, contractions, and rotation of the abdomen followed by the extension of one or both back paws were counted over the subsequent 30 min.

The extracts, fractions, and essential oils were tested in the following doses: EE_M_ and EE_C_—2000, 1000, and 500 mg kg^−1^; FH_C_—400 mg kg^−1^; DF_C_—240 mg kg^−1^; EAF_C_—1160 mg kg^−1^; AF_C_—480 mg kg^−1^; EO_C_—60, 200, 600 mg kg^−1^. Indomethacin was used at a dose of 10 mg kg^−1^. The control groups received the vehicle used in the solubilisation of each extract, fraction, or essential oil (10 mL kg^−1^), so there were three control groups that received either 10% propylene glycol in CMC gel, 10% DMSO in water, or only water.

#### 2.6.2. Anti-Inflammatory Activity by Testing Ear Oedema Induced by Croton Oil

Tests of ear oedema induced by croton oil were carried out according to Zanini et al. [40]. Groups of mice (*n* = 10) were treated (v.o.) with dexamethasone (2.0 mg kg^−1^), vehicle (10% propylene glycol in CMC gel, 10 mL kg^−1^), or EE_C_ (2000, 1000, and 500 mg kg^−1^). One hour after treatment, each animal received 20 *μ*L of a freshly prepared solution of croton oil (2.5% v/v) in acetone on the surface of the right ear. The left ear received the same volume of acetone. After 4 h, the animals were sacrificed and identical segments were taken from both ears. The formations and intensities of the oedema were expressed as the mean of differences in weight between the segments of the animals' ears: the smaller the weight difference, the greater the potential for inhibition.

### 2.7. Statistical Analysis

The antinociceptive effects of the different extracts, fractions, and essential oils were expressed as the means (±SEM) and are shown as percentages relative to the control group (vehicle). The significant differences between treated and control groups (vehicle) were assessed by an ANOVA and a multiple comparison by Tukey-Kramer. *P* values <0.05 were considered statistically significant. The data analysis used the application GraphPad Prism 3.0.

## 3. Results and Discussion

### 3.1. Phytochemical Analysis

The phytochemical analysis of the fractions obtained from the crude ethanol extracts of *P. pseudocaryophyllus* leaves of the citral and (*E*)-methyl isoeugenol chemotypes enabled the identification of pentacyclic triterpenes (lupeol, *α*-amyrin, *β*-amyrin, oleanolic acid, betulinic acid, and ursolic acid), flavonoids (quercetin 3-O-*α*-L-rhamnopyranoside, quercetin 3-O-*β*-glucopyranoside, kaempferol 3-O-*α*-L-rhamnopyranoside, quercetin 3-O-*α*-arabinofuranoside, quercetin 3-O-*α*-arabinopyranoside, quercetin 3-O-*β*-arabinopyranoside, and catechin), gallic acid, and ellagic acid (Table 2). These constituents, except ursolic acid, were found for the first time in this species. They were identified based on spectra of 1D and 2D ^1^H and ^13^C NMR (HSQC and HMBC) and with comparison with data in the literature (copies of the original spectra can be obtained from the corresponding author). The flavonoid quercitrin was also identified based on the mass spectrum and analysis by HPLC in comparison with an authentic sample.

The results from the qualitative and quantitative analysis of volatile oils of *P. pseudocaryophyllus*, with the volatile constituents listed in order of elution, are found in Table 3. A total of 31 compounds were identified, accounting for 94–100% of the volatile components. There was a predominance of phenylpropanoid derivatives (97.5%) among the volatile components of the (*E*)-methyl isoeugenol chemotype, and almost all of the oil consisted of (*E*)-methyl isoeugenol (93.9%). The essential oil of the citral chemotype consisted mainly of oxygenated mono- and sesquiterpenes (69.65% and 13.7%, resp.), and the monoterpene aldehydes neral and geranial, which are referred to as citral when their isomers are mixed, were the major components (27.59% and 36.49%, resp.).

### 3.2. Antimicrobial Activity

The MIC values of extracts, fractions, and essential oils, as well as citral (Sigma), are described in Table 4.  Table 5 shows the MIC of the semipurified substances and of vancomycin, gentamicin, and itraconazole, which were used as controls. The results were discussed taking into account the classification by Holetz et al. [41], which has also been adopted by other authors [42–44], where a MIC lower than 100 *μ*g mL^−1^ represents good antimicrobial activity; a MIC from 100 to 500 *μ*g mL^−1^ represents moderate antimicrobial activity; a MIC from 500 to 1000 *μ*g mL^−1^ represents weak activity; a MIC above 1000 *μ*g mL^−1^ suggests that the substance is inactive. EE_C_ showed the lower MIC compared to EE_M_ for Gram-positive bacteria, and both were inactive for Gram-negative bacteria (Table 4). EE_C_ was the most active for fungal strains, with a MIC between 7.8 and 62.5 *μ*g mL^−1^. The lowest MIC of this extract was found for the strains of *Cryptococcus* (MIC = 7.8–15.6 *μ*g mL^−1^), while EE_M_ showed good-to-moderate activity (MIC = 15.6–125 *μ*g mL^−1^) (Table 4) for the yeasts studied.

Regarding the fractions and semipurified substances, the dichloromethanic fractions of both chemotypes (DF_M_ and DF_C_) were the most active for Gram-positive bacteria (MIC = 250–500 *μ*g mL^−1^) and were less active for the fungal strains (MIC ≥ 500 *μ*g mL^−1^) (Table 4). The antibacterial activity of DF_C_ is due to substance **Ppc-2**, which had the greatest inhibitory effect on Gram-positive bacteria of the compounds studied (MIC = 31.2 *μ*g mL^−1^ for *S. aureus* ATCC 6538, *S. epidermidis* ATCC 12229, and *B. cereus* ATCC 14579; MIC = 62.5 *μ*g mL^−1^ for *S. aureus* ATCC 25923, *M. roseus* ATCC 1740, and *B. atrophaeus* ATCC 6633; MIC = 125 *μ*g mL^−1^ for *M. luteus* ATCC 9341) (Table 5). The phytochemical analysis showed that **Ppc-2** is oleanolic acid, a pentacyclic triterpene with antibacterial activity [45–47], which was especially effective against Gram-positive bacteria. The TLC analysis of the DF_M_ showed the presence of oleanolic, betulinic, and ursolic acids, which probably contributed to the observed antibacterial activity of this fraction [45–47]. **Ppm-4**, isolated from EAF_M_ and composed of quercitrin, afzelin, isoquercitrin, avicularin, guaijaverin, catechin, and gallic acid, showed activity against *C. neoformans* L2 and L1 (CIM = 62.5 *μ*g mL^−1^) but was not active against *Candida* (Table 5). Studies have shown that catechin and quercitrin, the main components of **Ppm-4**, inhibit the growth of some pathogenic fungi [48–50]. **Ppm-5**, obtained from the AF_M_, showed an activity profile similar to this fraction, except for *C. parapsilosis* 11A (Table 5). The phytochemical analysis carried out on **Ppm-5** verified the presence of gallic acid and its derivatives, which are known for their antimicrobial activities [51–53], as well as the presence of sugars. The **Ppc-4** isolated from EAF_C_, which was composed of quercitrin, showed moderate inhibition of *Candida* strains (MIC = 250 *μ*g mL^−1^) and good-to-moderate inhibition of the strains of *Cryptococcus *(MIC = 62.5 to 250 *μ*g mL^−1^) (Table 5), partly contributing to the antifungal activity of this fraction. **Ppc-5**, which was also isolated from fraction EAF_C_, consisted of a mixture of flavonoids of which the major component is quercitrin and did not show activity under the conditions tested. **Ppc-6** contributed in part to the antifungal activity of AF_C_, with MICs ranging from 62.5 to 125 *μ*g mL^−1^ for *Candida *strains (Table 5). The phytochemical analysis of **Ppc-6** showed ellagic acid to be the major component. Similar results for *Candida *spp., due to ellagic acid, were reported by Silva et al. [54]. These findings are promising in the search for new options against infections caused by *Candida* spp. and *Cryptococcus* spp., particularly with the continuous increase of opportunistic fungal resistance to available treatments, the emergence of rare species of *Candida *[55], and the increasing number of infections by *Cryptococcus neoformans* and *C. gattii* [56].

The essential oil of the citral chemotype (EO_C_) showed good activity (MIC = 62.5 *μ*g mL^−1^) against* B. cereus *ATCC 14579 and moderate activity (MIC = 125–250 mL g *μ*g mL^−1^) against the remaining Gram-positive bacteria; in some cases, this essential oil showed better inhibition than citral (MIC = 125–250 *μ*g mL^−1^) (Table 4). Citral, the major component of the EO_C_, is a monoterpene aldehyde mixture of the isomers neral and geranial. Aldehydes, such as formaldehyde and glutaraldehyde, are known to have strong antimicrobial activity, and several researchers have demonstrated the antimicrobial effect of citral [57–62]. It is suggested that the aldehyde group conjugated to the carbon-carbon double bond, which is present in neral and geranial, provides a highly electronegative chemical structure that may explain its activity [57]. The EO_M_ was inactive against bacteria under the conditions tested. Its major component, the phenylpropanoid (*E*)-methyl isoeugenol, comprises almost all of the oil (93.9%). Structurally, (*E*)-methyl isoeugenol differs from eugenol because it has a methoxyl group at the C1 position of the ring instead of a hydroxyl group, and the double bond in its propenyl group is in a different position. The absence of the hydroxyl group in the phenolic structure of (*E*)-methyl isoeugenol may have contributed to the lack of activity of this compound. Griffin et al. [63] observed that the methylation of the hydroxyl group in eugenol, producing methyleugenol, resulted in a loss of activity for Gram-negative bacteria.

The EO_C_ showed good activity against *Cryptococcus* (MIC = 15.6 *μ*g mL^−1^ to 62.5 *μ*g mL^−1^), but the EO_M_ showed moderate activity against strains of *Cryptococcus* (MIC = 125 *μ*g mL^−1^ to 250 *μ*g mL^−1^) and showed no activity for species of *Candida *obtained from clinical isolates (MIC > 500 *μ*g mL^−1^) (Table 4). The structure-activity relationship of the components of certain essential oils has been researched in phytopathogenic fungi, and there is little information on fungal pathogens in humans. For example, carbonyl *α*,*β*-unsaturated compounds, such as the monoterpenic aldehydes neral and geranial, have strong antifungal activity [27, 64, 65], similar to their antibacterial activity. Little information regarding the antifungal mechanism of action of these aldehydes is available. The evaluation of the effects of citral on the membranes, organelles, and intracellular macromolecules of *Aspergillus flavus* spores has shown that citral damages the cell walls and membranes of spores, reducing their elasticity [66].

### 3.3. Antinociceptive and Anti-Inflammatory Screening

**Figure 1:**
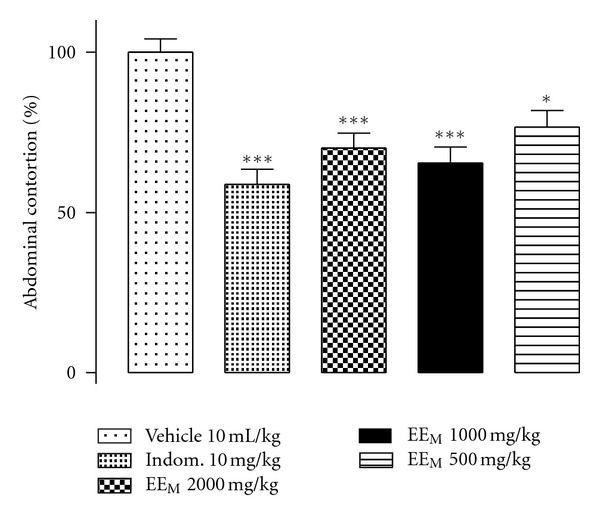
EE_M_ antinociceptive effect in the abdominal contortion model induced by acetic acid (1.2% v/v, i.p.) in mice previously treated with EE_M_ (2000, 1000, and 500 mg kg^−1^, v.o). Vehicle (CMC + propylene glycol) 10 mL kg^−1^, v.o. Indomethacin was used as positive control (10 mg kg^−1^, v.o). Each column shows the mean (±SEM) of number of contortions in percentages relative to control (vehicle). *Indicates the significance level compared to the control group (vehicle). *n* = 10 mice. *P < 0.05, ***P < 0.001.

The EE_M_ (v.o. 2000, 1000, and 500 mg kg^−1^) significantly reduced the number of abdominal contortions caused by the intraperitoneal injection of acetic acid in mice compared to animals that received only vehicle (control group) (Figure 1). There was no significant difference observed between the analgesic effects of the three doses used (data not shown).

**Figure 2:**
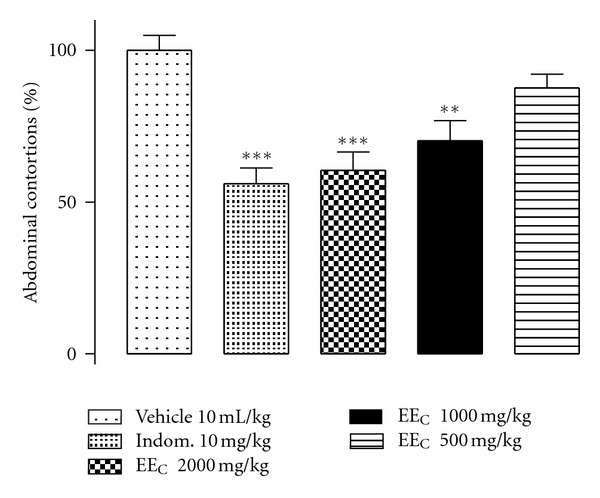
EE_C_ antinociceptive effect in the abdominal contortion model induced by acetic acid (1.2% v/v, i.p.) in mice previously treated with EE_C_ (2000, 1000, and 500 mg kg^−1^, v.o). Vehicle (CMC + propylene glycol) 10 mL kg^−1^, v.o. Indomethacin was used as positive control (10 mg kg^−1^, v.o). Each column shows the mean (±SEM) of number of contortions in percentages relative to control (vehicle). *Indicates the significance level compared to the control group (vehicle). *n* = 10 mice. *P < 0.05, **P < 0.01, ***P < 0.001.

The EE_C_ (v.o. 2000 and 1000 mg kg^−1^) was able to inhibit, significantly and in a dose-dependent manner, the abdominal contortions in mice induced by the intraperitoneal acetic acid compared with the control group and showed no significant difference compared to the group of animals treated with indomethacin (10 mg kg^−1^) (Figure 2).

**Figure 3:**
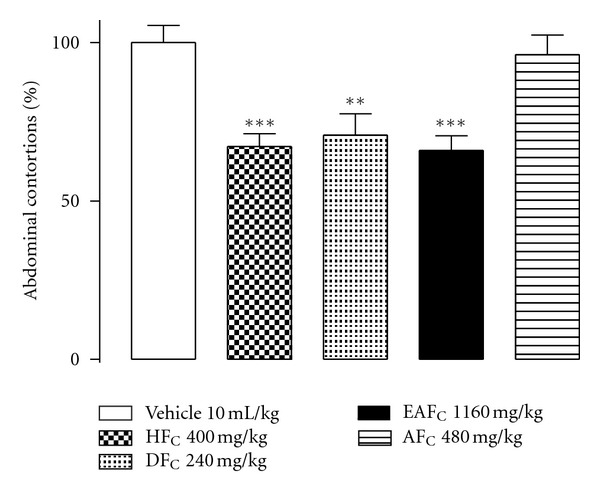
Antinociceptive effect of fractions in the abdominal contortion model induced by acetic acid (1.2% v/v, i.p.) in mice previously treated with HF_C_ (400 mg kg^−1^, v.o), DF_C_ (240 mg kg^−1^, v.o), EAF_C_ (1160 mg kg^−1^, v.o), and AF_C_ (480 mg kg^−1^, v.o). Vehicle (water + DMSO 10%) 10 mL kg^−1^, v.o. Each column shows the mean (±SEM) of number of contortions in percentages relative to control (vehicle). *Indicates the significance level compared to the control group (vehicle). *n* = 10 mice. *P < 0.05, **P < 0.01, ***P < 0.001.

Given the dose-dependent antinociceptive effect shown by EE_C_ (Figure 2), this extract was selected to perform the screening of the antinociceptive activity of its fractions and essential oils, as well as to evaluate its anti-inflammatory activity by testing ear oedema induced by croton oil. HF_C_ (400 mg kg^−1^), DF_C_ (240 mg kg^−1^), and EAF_C_ (1160 mg kg^−1^) significantly decreased the number of abdominal contortions induced by the intraperitoneal injection acetic acid compared with the control group. However, there was no significant difference in the antinociceptive effect shown by the three fractions. The group of animals that received AF_C_ (480 mg kg^−1^) showed a number of contortions similar to that of the group that received vehicle (Figure 3).

**Figure 4:**
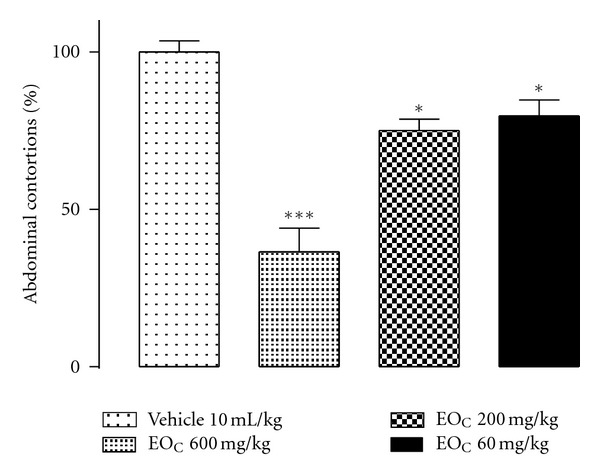
EO_C_ antinociceptive effect of abdominal contortion model induced by acetic acid (1.2% v/v, i.p.) in mice previously treated with EO_C_ (600, 200, and 60 mg kg^−1^, v.o). Vehicle (water + DMSO 10%) 10 mL kg^−1^, v.o. Each column shows the mean (±SEM) of number of contortions in percentages relative to control (vehicle). *Indicates the significance level compared to the control group (vehicle). *n* = 10 mice. *P < 0.05, ***P < 0.001.

The EO_C_ at doses of 60, 200, and 600 mg kg^−1^ (v.o.) showed significant dose-dependent inhibitory effects on the abdominal contortions induced by intraperitoneal acetic acid in mice compared to the control group (Figure 4).

**Figure 5:**
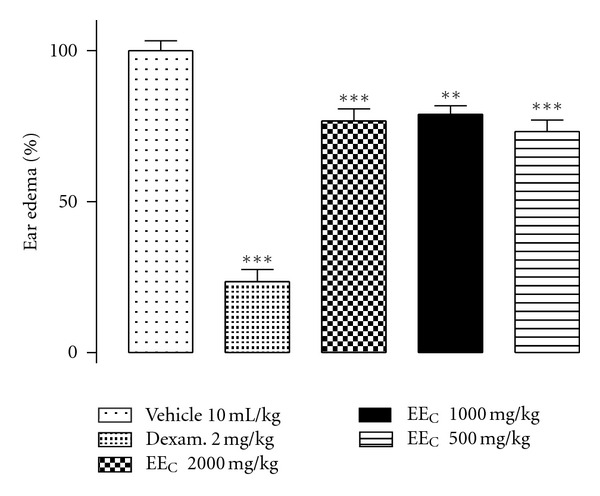
EE_C_ anti-inflammatory effect under ear oedema induced by croton oil (2.5% v/v) in mice previously treated with EE_C_ (2000, 1000, and 500 kg^−1^, v.o). Control (CMC + propylene glycol) 10 mL kg^−1^, v.o. Dexamethasone was used as positive control (2 mg kg^−1^, v.o). Each column expresses the mean (±SEM) of the difference of the weights of ear segments in percentages relative to control (vehicle). *Indicates the significance level compared to the control group (vehicle). *n* = 10 mice. **P < 0.01, ***P < 0.001.

The ear oedema induction test showed that the EE_C_ has antiedematogenic activity that is significantly greater than that of the control at all doses tested (Figure 5). Pretreatment with EE_C_ (2000, 1000, and 500 mg kg^−1^) reduced the oedema from 14.3 ± 0.4 mg (control) to 10.9 ± 0.5, 11.3 ± 0.4, and 10.5 ± 0.5 mg, respectively.

The results obtained from this study showed that both EE_M_ and EE_C_ have antinociceptive activity. It was not possible to infer whether this action involves peripheral and/or central mechanisms, as the model of abdominal contortions induced by the intraperitoneal injection of acetic acid in mice is sensitive to analgesic substances that act centrally and/or peripherally and that show a wide range of mechanisms of action [39]. Similar results were observed in other species of the genus *Pimenta *[29, 30, 32].

The mixture of triterpenes lupeol and *α*- and *β*-amyrin isolated from HF_C_ could be accountable for the observed activity. Several *in vivo* experiments have shown that, among the pharmacological activities attributed to these compounds, analgesic and anti-inflammatory effects are predominant [31, 67–72].

Oleanolic acid, which is widely distributed in the vegetable kingdom, has strong anti-inflammatory effects [73] that may have contributed to reducing the number of abdominal contortions induced by chemical stimulation in mice, as observed for the DF_C_.

Quercetin, quercitrin, and kaempferol have anti-inflammatory activities [74, 75], which may also have contributed for the analgesic activity of EAF_C_ observed in this study.

Data from the literature report the antinociceptive activity of citral [76, 77], a major component of the EO_C_. Moreover, sedative, anxiolytic, and anticonvulsant effects were observed from the essential oils of *Cymbopogon citratus *in mice through the use of motor activity tests (“rota-rod” and “open-field”), a hypnosedative activity test (sleep induced by barbiturate), an anxiolytic action test (“plus-maze” and “light-dark box”), and an anticonvulsant action test (seizures chemically induced by pentylenetetrazole) [78]. Therefore, the inhibition by the EO_C_ of the contortions induced by chemical stimulation in mice in this study may be due to both peripheral and central mechanisms. However, the sedative and relaxing effects of citral, as well as the increase in sleep time induced by barbital, especially at high doses (200 mg kg^−1^) [79], may have exerted a greater influence on reducing the number of contortions than the analgesic effects themselves.

## 4. Conclusion

Until now, no systematic phytochemical and biological study on citral and the (*E*)-methyl isoeugenol chemotypes of *P. pseudocaryophyllus* had been reported. Among the isolated substances, oleanolic acid obtained from the dichloromethane fraction of the citral chemotype showed the best profile of antibacterial activity against the Gram-positive microorganisms used in this research (MIC = 31.2 to 125 *μ*g mL^−1^), followed by the essential oil of the citral chemotype, which showed good activity (MIC = 62.5 g *μ*g mL^−1^) against *B. cereus* ATCC 14579 and moderate activity (MIC = 125–250 *μ*g mL^−1^) against the other Gram-positive microbes.

The extracts, fractions, and essential oils from *P. pseudocaryophyllus* leaves showed several levels of antifungal activity against *Candida* spp. and *Cryptococcus* spp. The antifungal activity shown by the ethyl acetate and aqueous fractions from both chemotypes, especially against *Candida albicans* (MIC = 31.2 to 62.5 *μ*g mL^−1^) and *C. parapsilosis* (MIC = 15.6 to 62.5 *μ*g mL^−1^), showed the potential of this species as a source of new antifungal alternatives.

In the models of abdominal contortions induced by acetic acid and ear oedema induced by croton oil in mice, the crude extract of the citral chemotype showed antinociceptive and anti-inflammatory effects, respectively. These effects may be related to the presence of the pentacyclic triterpenes lupeol, *α*-amyrin, and *β*-amyrin and the flavonoids quercetin, quercitrin, and afzelin.

## Figures and Tables

**Table 1:** Microorganisms used in determining the minimum inhibitory concentration (MIC).

Microorganisms	ATCC	Clinic isolated
Gram (+) bacteria		
*Staphylococcus * *aureus *	6538	
*Staphylococcus * *aureus *	25923	
*Staphylococcus * *epidermidis *	12229	
*Micrococcus * *luteus *	9341	
*Micrococcus * *roseus *	1740	
Sporulated Gram (+)		
*Bacillus * *cereus *	14579	
*Bacillus * *atrophaeus *	6633	
Gram (−) bacteria		
*Escherichia * *coli *	8739	
*Escherichia * *coli *	11229	
*Enterobacter * *aerogenes *	13048	
*Enterobacter * *cloacae *		HMA/FT502
*Serratia * *marcescens *	14756	
*Salmonella * spp.	19430	
*Pseudomonas * *aeruginosa *	27483	
*Pseudomonas * *aeruginosa *		SPM1
Fungi		
*Candida * *albicans *		02
*Candida * *parapsilosis *		11-A
*Candida * *parapsilosis *	22019	
*Cryptococcus * *neoformans *	28957	
*Cryptococcus * *neoformans *		L2
*Cryptococcus * *gattii *		L1

**Table 2:** Substances identified in *Pimenta pseudocaryophyllus* citral and (*E*)-methyl isoeugenol chemotypes.

Substances semipurified in citral chemotype (mg)	Substances semipurified in (*E*)-methyl isoeugenol chemotype (mg)	Compounds identified (approximated amount*—mg)	References
**Ppc-1** (133.5)		Lupeol (60.7), *α*-amyrin (20.2), and *β*-amyrin (52.6)	[14]
**Ppc-2** (22.6)		Oleanolic acid	[14]
**Ppc-3** (59.7)		Quercetin	[15, 16]
**Ppc-4** (18.7)		Quercetin 3-O-*α*-L rhamnopyranoside—quercitrin	[17]
**Ppc-5** (73.2)		Quercetin 3-O-*α*-L-rhamnopyranoside—quercitrin (46.2), quercetin 3-O-*β*-glycopyranoside—isoquercitrin (6.6), kaempferol 3-O-*α*-L-rhamnopyranoside—afzelin (6.6), and catechin (13.8)	[17–19]
**Ppc-6** (83.1)		Gallic acid, ellagic acid, and not identified derivatives	[15, 20]
	**Ppm-1** (106.2)	(*E*)-methyl isoeugenol	[21]
	**Ppm-2** (75.8)	Lupeol (47.4), *α*-amyrin (9.5), and *β*-amyrin (18.9)	[14]
	**Ppm-3** (36.9)	Oleanolic acid (25.8), ursolic acid (7.4), and betulinic acid (3.7)	[14, 22]
	**Ppm-4** (273.4)	Quercetin 3-O-*α*-L-rhamnopyranoside—quercitrin (88.3), quercetin 3-O-*β*-glycopyranoside—isoquercitrin (8.8), kaempferol 3-O-*α*-L-rhamnopyranoside—afzelin (17.6), quercetin 3-O-*α*-arabinofuranoside—avicularin (8.8), quercetin 3-O-*α*-arabinopyranoside—guaijaverin (8.8), quercetin 3-O-*β*-arabinopyranoside (8.8), gallic acid (2.5), and catechin (105.8).	[15, 17–19, 23]
	**Ppm-5** (203.6)	Gallic acid and not identified derivatives	[15]

*Acquired from ^1^H NMR data.

**Table 3:** Percentage chemical composition of essential oils from leaves of *P. pseudocaryophyllus* (*E*)-methyl isoeugenol and citral chemotypes.

Component	RI	MC	CC
(1) (2*E*)-Hexenol	846		0.24
(2) UC	918		0.05
(3) *α*-Tujene	929		0.46
(4) Allyl isovalerate	938		0.49
(5) UC	947		0.47
(6) *β*-Pinene	973		1.41
(7) 6-Methyl-5-hepten-2-one	980		0.71
(8) Dehydro-1.8-cineole	987		0.23
(9) Linalool	1096		1.02
(10) *exo*-Isocitral	1139		0.27
(11) (*Z*)-Isocitral	1159		0.36
(12) UC	1175		0.28
(13) (*E*)-Isocitral	1177		0.51
(14) UC	1203		0.19
(15) Nerol	1224		1.05
(16) Neral	1237		27.59
(17) Geraniol	1250		2.13
(18) Geranial	1266		36.49
(19) UC	1332		1.61
(20) UC	1358		0.49
(21) UC	1368		2.1
(22) *α*-Copaene	1373	0.4	1.03
(23) *β*-Bourbonene	1381		0.7
(24) *β*-Elemene	1389		0.31
(26) (*E*)-Caryophyllene	1416	1.7	1.55
(27) *α*-Copaene	1425		0.27
(28)Aromadendrene	1435		0.55
(29) *α*-Humulene	1450		0.27
(30) (*Z*)-Methyl isoeugenol	1451	2.0	
(31) *Allo*-Aromadendrene	1457		0.57
(32) *γ*-Muurolene	1472		0.36
(33) (*E*)-Methyl isoeugenol	1494	93.9	
(34) *γ*-Cadinene	1510		1.15
(35) *δ*-Cadinene	1520	0.4	0.32
(36) UC	1548		0.77
(37) Spathulenol	1573		3.75
(38) Caryophyllene oxide	1578		8.88
(39) Humulene epoxide II	1604		1.07

Monoterpene hydrocarbons			1.87
Oxygenated monoterpenes			69.65
Sesquiterpene hydrocarbons		2.5	7.08
Oxygenated sesquiterpenes			13.7
Other			1.44
Phenols		97.5	

Yield (%, v/p)		0.8	0.8

RI: calculated retention index. MC: *P.* *pseudocaryophyllus* (*E*)-methyl isoeugenol chemotype. CC: *P.* *pseudocaryophyllus* citral chemotype. UC: unknown component.

**Table 4:** Minimum inhibitory concentration (*μ*g mL^−1^) of extracts, fractions, and essential oils from leaves of *P.* *pseudocaryophyllus* (*E*)-methyl isoeugenol and citral chemotypes, and of the citral substance.

Microorganisms	EE_M_	HF_M_	DF_M_	EAF_M_	AF_M_	EO_M_	EE_C_	HF_C_	DF_C_	EAF_C_	AF_C_	EO_C_	Citral
													(Sigma)
Gram (+) bacteria													
*S*. *aureus *	1000	1000	500	1000	1000	>1000	500	>1000	250	500	1000	250	125
ATCC 6538													
*S*. *aureus *	1000	1000	500	1000	1000	>1000	500	>1000	500	1000	1000	125	125
ATCC 25923													
*S*. *epidermidis *	1000	1000	250	1000	1000	>1000	1000	1000	250	500	1000	125	250
ATCC 12229													
*M*. *luteus *	1000	1000	250	1000	1000	>1000	500	>1000	250	500	1000	125	125
ATCC 9341													
*M*. *roseus *	1000	1000	500	500	1000	>1000	500	1000	500	500	1000	125	125
ATCC 1740													
*B*. *cereus *	1000	1000	250	1000	500	>1000	500	1000	250	500	1000	62.5	125
ATCC 14579													
*B*. *atrophaeus *	1000	1000	500	1000	1000	>1000	500	>1000	500	500	1000	125	125
ATCC 6633													
Gram (−) bacteria													
*E*. *coli *	>1000	>1000	>1000	1000	>1000	>1000	>1000	>1000	>1000	1000	1000	>1000	>1000
ATCC 8739													
*E*. *coli *	>1000	>1000	>1000	1000	>1000	>1000	>1000	>1000	>1000	1000	1000	>1000	1000
ATCC 11229													
*E*. *aerogenes *	>1000	>1000	>1000	1000	>1000	>1000	>1000	>1000	>1000	1000	>1000	>1000	>1000
ATCC 13048													
*E*. *cloacae *	>1000	>1000	>1000	1000	>1000	>1000	>1000	>1000	>1000	1000	1000	>1000	1000
HMA/FTA502													
*S*. *marcescens *	>1000	>1000	>1000	>1000	>1000	>1000	>1000	>1000	>1000	1000	>1000	>1000	>1000
ATCC 14756													
*Salmonella *spp.	1000	1000	1000	1000	1000	>1000	1000	>1000	1000	1000	1000	>1000	500
ATCC 19430													
*P*. *aeruginosa *	1000	1000	1000	1000	1000	>1000	1000	1000	1000	500	1000	>1000	1000
ATCC 27483													
*P*. *aeruginosa *	1000	1000	1000	1000	500	>1000	1000	1000	1000	1000	1000	>1000	1000
SPM1													
Fungi													
*C*. *albicans *02	125	62.5	>500	31.2	62.5	>500	62.5	500	250	31.2	62.5	500	125
*C*. *parapsilosis *11A	62.5	62.5	500	15.6	31.2	>500	31.2	250	250	31.2	31.2	500	250
*C*. *parapsilosis *	62.5	62.5	500	31.2	62.5	>500	31.2	250	250	31.2	31.2	500	125
ATCC 22019													
*C*. *neoformans* var.	15.6	15.6	>500	>500	15.6	125	15.6	250	500	7.8	15.6	15.6	7.8
*neoformans *													
ATCC 28957													
*C*. *neoformans* var.	125	31.2	500	>500	7.8	250	7.8	62.5	500	15.6	>500	62.5	15.6
*neoformans *L2													
*C*. *neoformans* var.	125	31.2	>500	>500	31.2	250	15.6	500	500	15.6	>500	62.5	31.2
*gatti *L1													

EE_M_: ethanol extract of the (*E*)-methyl isoeugenol chemotype. HF_M_: hexane fraction of the (*E*)-methyl isoeugenol chemotype. DF_M_: dichloromethane fraction of the (*E*)-methyl isoeugenol chemotype. EAF_M_: ethyl acetate fraction of the (*E*)-methyl isoeugenol chemotype. AF_M_: aqueous fraction of the (*E*)-methyl isoeugenol chemotype. EO_M_: essential oil of the (*E*)-methyl isoeugenol chemotype. EE_C_: ethanol extract of the citral chemotype. HF_C_: hexane fraction of the citral chemotype. DF_C_: dichloromethane fraction of the citral chemotype. EAF_C_: ethyl acetate fraction of the citral chemotype. AF_C_: aqueous fraction of the citral chemotype. EO_C_: essential oil of the citral chemotype.

**Table 5:** Minimum inhibitory concentration (*μ*g mL^−1^) of semipurified substances of *P*.  *pseudocaryophyllus*.

Microorganisms	Ppc-2	Ppc-4	Ppc-5	Ppc-6	Vanc.	Genta.	Ppm-1	Ppm-3	Ppm-4	Ppm-5	Itrac.
Gram (+) bacteria											
*S*. *aureus *	31.2	>500	1000	NC	2	NC	NC	NC	NC	NC	NC
ATCC 6538											
*S*. *aureus *	62.5	>500	>1000	NC	1	NC	NC	NC	NC	NC	NC
ATCC 25923											
*S*. *epidermidis *	31.2	500	>1000	NC	1	NC	NC	NC	NC	NC	NC
ATCC 12229											
*M*. *luteus *	125	500	>1000	NC	0.25	NC	NC	NC	NC	NC	NC
ATCC 9341											
*M*. *roseus *	62.5	>500	1000	NC	0.5	NC	NC	NC	NC	NC	NC
ATCC 1740											
*B*. *cereus *	31.2	500	>1000	NC	2	NC	NC	NC	NC	NC	NC
ATCC 14579											
*B*. *atrophaeus *	62.5	>500	1000	NC	0.25	NC	NC	NC	NC	NC	NC
ATCC 6633											
Gram (−) bacteria											
*E*. *coli *	>1000	>500	>1000	NC	NC	8	NC	NC	NC	NC	NC
ATCC 8739											
*E*. *coli *	>1000	>500	>1000	NC	NC	2	NC	NC	NC	NC	NC
ATCC 11229											
*E*. *aerogenes *	>1000	>500	>1000	NC	NC	0.125	NC	NC	NC	NC	NC
ATCC 13048											
*E*. *cloacae *	>1000	>500	>1000	NC	NC	4	NC	NC	NC	NC	NC
HMA/FTA502											
*S*. *marcescens *	>1000	>500	>1000	NC	NC	4	NC	NC	NC	NC	NC
ATCC 14756											
*Salmonella* spp.	>1000	>500	>1000	NC	NC	2	NC	NC	NC	NC	NC
ATCC 19430											
*P*. *aeruginosa *	>1000	>500	>1000	NC	NC	4	NC	NC	NC	NC	NC
ATCC 9027											
*P*. *aeruginosa *	>1000	>500	>1000	NC	NC	4	NC	NC	NC	NC	NC
SPM1											
Fungi											
*C*. *albicans *02	>600	250	>500	62.5	NC	NC	500	>500	>500	31.2	1
*C*. *parapsilosis* 11A	>600	250	>500	125	NC	NC	500	>500	>500	>1000	1
*C*. *parapsilosis *	>600	250	>500	125	NC	NC	500	>500	>500	31.2	1
ATCC 22019											
*C*. *neoformans* var.	>600	62.5	>500	>500	NC	NC	250	>500	500	15.6	2
*neoformans *											
ATCC 28957											
*C*. *neoformans* var.	>600	62.5	>500	500	NC	NC	250	500	62.5	3.9	2
*neoformans* L2											
*C*. *neoformans* var.	>600	250	>500	500	NC	NC	250	500	62.5	3.9	2
*gatti *L1											

**Ppc-2**: oleanolic acid. **Ppc-4**: quercitrin. **Ppc-5**: quercitrin, isoquercitrin, afzelin, and catechin. **Ppc6**: ellagic acid, gallic acid, and sugars. **Ppm-1**: (*E*)-methyl isoeugenol. **Ppm-3**: oleanolic acid, ursolic acid, and betulinic acid. **Ppm-4**: quercitrin, isoquercitrin, afzelin, catechin, avicularin, guaijaverin, and gallic acid. **Ppm-5**: gallic acid and sugars. Vanc.: Vancomycin (32 *μ*g mL^−1^). Gent.: gentamicin (128 *μ*g mL^−1^).Itrac.: itraconazole (16 *μ*g mL^−1^). NC: not carried out.
